# Factors influencing pregnant women’s decision to accept or decline prenatal screening and diagnosis – a qualitative study

**DOI:** 10.1007/s12687-024-00746-3

**Published:** 2024-11-01

**Authors:** Ellen Ternby, Ove Axelsson, Charlotta Ingvoldstad Malmgren, Susanne Georgsson

**Affiliations:** 1 Department of Women’s and Children’s Health, Uppsala University, Uppsala, Sweden; 2 Centre for Clinical Research Sörmland, Uppsala University, Eskilstuna, Sweden; 3 Department of Molecular Medicine and Surgery, Karolinska Institutet, Stockholm, Sweden; 4 Centre for Fetal Medicine, Karolinska University Hospital, Stockholm, Sweden; 5 Centre for Research Ethics and Bioethics, Uppsala University, Uppsala, Sweden; 6 The Swedish Red Cross University, Stockholm, Sweden

**Keywords:** Information, Informed choice, Genetic counseling, Decision making, Prenatal diagnosis, Chromosome aberrations

## Abstract

**Background and Objective:** Prenatal diagnosis for chromosomal anomalies is frequently used worldwide. It is important that pregnant women receive adequate counselling to make informed decisions regarding prenatal diagnosis. The aim of this study was to explore what factors influence pregnant women’s decision-making process when accepting or declining prenatal screening and diagnosis.  **Methods: **A qualitative study using inductive qualitative content analysis. Individual, semi-structured phone interviews were carried out during a five-month period in 2016–2017 with 24 pregnant women in the first trimester, living in a medium-sized Swedish city. **Findings:** Two main themes emerged: (1)*“Individual factors - The women’s experiences*,* perceptions and values”* with three categories “Attitude towards anomalies”, “Worry and need for reassurance”, “Self-perceived risk” and (2)*“External factors - The women’s perception of the test and others’ views”* with two categories “Test characteristics” and “Influence from others”.  **Conclusions:** Pregnant women’s decision-making process regarding prenatal tests is multidimensional, affected by both individual factors such as experiences, perceptions and values, and external factors such as test characteristics and influence from others. Information about both test characteristics and the conditions tested is of help pregnant women in the decision-making process since it provides a better understanding of how having a child with the condition in question can affect them and their family. It is important that healthcare professionals giving information about and offering prenatal tests for chromosomal anomalies are aware of how their attitudes can influence women’s decisions.

**Supplementary Information:**

The online version contains supplementary material available at 10.1007/s12687-024-00746-3.

## Introduction

Approximately 70% of pregnant women in Sweden undergo prenatal screening for chromosomal anomalies (CA) with a combined ultrasound and biochemistry test (CUB test) (Skogsdal et al. 2022). The screening is offered as part of the public antenatal care, but how it is offered varies between regions (The National Board of Health and Welfare (Socialstyrelsen) 2022). If a CUB test indicates a high probability of a CA, Non-Invasive Prenatal Testing (NIPT) or an invasive test are offered as a second-tier test.

According to a recent review of quantitative research (Di Mattei et al. 2021) there seems to be different aspects affecting pregnant women’s decisions regarding prenatal screening and diagnosis (PND). They describe an individual level including demographic, psychological and clinical aspects; a relational level affected by family and society; and a contextual level including test characteristics and received information. A previous review, including both quantitative and qualitative studies, has shown similar findings, where test characteristics as well as individual factors such as age, anxiety, personal or professional experience of and attitude towards disability and termination of pregnancy (TOP) were factors affecting the decision-making process (Crombag et al. 2013). From research in Sweden and Denmark, it is known that common reasons for undergoing PND are age, the wish to gain as much information as possible about the fetus, worry and the wish for reassurance of a healthy fetus (Miltoft et al. 2018; Sahlin et al. 2016; Ternby et al. 2015, 2016). Common reasons for abstaining from PND were the risk of miscarriage if having an invasive test and not seeing TOP as an option (Ternby et al. 2016). Another aspect known to influence women’s decisions is their partners, since many (but not all) parents-to-be, strive for a joint decision-making (Damman et al. 2023; Laberge et al. 2019; Miltoft et al. 2018; Sahlin et al. 2016). Most studies have focused on a few, but not all the mentioned aspects in one study, which may not provide a complete overall picture. Our qualitative approach can provide a broader picture and a deeper understanding of the different factors that influence the decision-making, and how they interplay, filling in this gap in the literature.

Previous research has indicated that the information given to pregnant women about PND and conditions screened for, is not always sufficient (Nykänen et al. 2017; Ternby et al. 2015, 2016) and that women do not always have sufficient knowledge or make informed decisions (Lewis et al. 2017; McCoyd 2013; van der Meij et al. 2022; Schoonen et al. 2012). Better knowledge concerning PND among pregnant women seem to result in higher levels of wellbeing and less anxiety, as well as less decisional conflict and regret (Dahl et al. 2011; Lo et al. 2019). In addition, women satisfied with their received counselling tend to experience less decisional conflict and less subsequent decisional regret (Hartwig et al. 2019). Thus, there is a need for better counselling and improved information prior to the offer of PND. The aim of the present study is to examine what factors influence women´s decisions to undergo or decline PND. Such knowledge can guide healthcare professionals as to what information should be included in counselling.

## Methods

### Study design

A qualitative design was chosen to explore subjective views and experiences and thus give a deeper understanding of this complex subject, which is more difficult to attain through a quantitative study design. Semi-structured, individual telephone interviews were conducted with pregnant women and data was analysed with inductive content analysis (Graneheim et al. 2017; Graneheim and Lundman 2004). The COREQ checklist was used as guidance (Tong et al. 2007).

### Recruitment and participants

Almost all pregnant women in Sweden attend publicly funded antenatal care, with regular check-ups with a midwife during pregnancy. Pregnant women meeting the inclusion criteria were asked about participation when booking their first appointment with their midwife at an antenatal care clinic in Sweden. Inclusion criteria were women pregnant in the first trimester, age 18 or older with a good command of the Swedish language. If consenting, they were called up by the first author (ET) who gave verbal and written information about the study and then interviewed them on a separate occasion. No records were kept of non-participants. In total, 24 pregnant women (gestational week 6–11) participated in telephone interviews before undergoing PND. Most women (21/24) had not yet met with the midwife and thus not received information about PND at the time of the interview. By the twentieth interview, recurring similarities and patterns were noticed by the interviewer, indicating data saturation (Rahimi and Khatooni 2024). The following four interviews confirmed that no new themes or subjects emerged and that *data saturation* had been reached. Inclusion of participants was therefore ended. During the data analysis process, *code and thematic saturation* (i.e. no new codes or themes, or relationships between them emerge) as well as *meaning saturation* (i.e. no new information about the meaning of codes or themes and their relationships emerge) was confirmed, in addition to the *data saturation* reached during the interviewing process (Rahimi and Khatooni 2024).

### Data collection

An overview of the current peer reviewed literature was performed, followed by discussions by the research team with clinical experience as an obstetrician (OA), a midwife (SG), a genetic counsellor (CIM) and a physician training in obstetrics and gynaecology (ET). Based on this, a semi-structured interview guide was developed. The use of a semi-structured interview guide was chosen to ensure that the same subjects would be discussed while maintaining flexibility during the interviews. The guide (Online Resource) covered different aspects of pregnant women’s informational needs regarding PND, their decision-making process, how they perceived healthcare professionals’ attitudes during consultations and their own attitudes towards fetal anomalies and Down syndrome. Data collection was carried out during October 2016- March 2017. The first four interviews were pilot interviews to test the guide. The research team evaluated the use of the interview guide in the pilot interviews and concluded it worked well. The guide ensured that all subjects relevant to the aim of the study were discussed, when complimented by follow-up questions based on how the interviews developed. Since no changes were made to the guide or study set up, the pilot interviews were included in the study. Each participant was interviewed by the first author (ET) on one occasion. The duration of each interview ranged from 15 to 37 min (mean 25). The participants chose the time and date for the interview.

### Data analysis

Interviews were made on speaker phone and audio recorded with an external smartphone and transcribed verbatim in Swedish. Due to delays in transcription and analyses, the transcripts were not returned to participants for the opportunity to provide feedback on the findings. Inductive content analysis (Graneheim et al. 2017; Graneheim and Lundman 2004) was performed by the first author, monitored by one of the co-authors (SG). In order to get a sense of the whole, the transcribed interviews were reread several times. To organize and analyse the qualitative data, the software tool Nvivo (version 11.4.3) was used. Meaning units relevant to the decision-making process regarding PND were identified and condensed. In the next step the condensed meaning units were abstracted into codes. The codes were then sorted into subcategories and categories, eventually resulting in themes. Categories and themes were not identified in advance but derived from the coding process. The results were discussed continuously with all authors to reach consensus on the interpretation. All analyses were performed in Swedish to assure no loss of nuances or meaning. The quotes used in this paper were translated into English by a professional translator.

### Ethical considerations

The study was approved by the Regional Ethical Review Board at the Medical Faculty of Uppsala University (Dnr 2015/227). Due to difficulties in recruitment, adjustments were made (i.e. switching to a region with better conditions for successful recruitment and switching from group interviews to individual interviews) and three supplementary applications were approved by the same board (Dnr 2015/227/1, 2015/227/2, 2015/227/3). The participants received both written and verbal information about the study. They were informed that participation was optional, that participation would not affect their care, and that they could withdraw at any time. Neither the interviewer (ET) nor any of the other researchers were involved in the care the participants received prior to or after the study. Participants were given time for reflection between receiving information and their participation in the interview. Verbal consent was given before the interview. The funders of this project were not involved in study design, data collection, data analysis, manuscript preparation or publication decisions.

## Results

The women interviewed were both nulliparous and parous, had diverse sociodemographic backgrounds and were between 25 and 36 years old. Their educational level ranged from basic schooling to university education (Table 1). None had had previous PND, except for first and/or second trimester ultrasound in previous pregnancies.

**Table 1:** Background data of the 24 participants

Background characteristic	*N* (%)
**Age**	
< 30	11 (45.8)
30–35	12 (50.0)
> 35	1 (4.2)
**Parity**	
Nulliparous	15 (62.5)
Parous	9 (37.5)
**Education**	
Upper secondary school	7 (29.2)
University/College	14 (58.3)
Unknown	3 (12.5)
**Experience of persons with anomalies** ^**a**^	
From family/friends	5 (20.8)
From work	6 (25.0)
From family member’s/friend’s work	2 (8.3)
No experience	11 (45.9)

^a^Personal experience from knowing an individual with an anomaly or disability or having been in contact with an individual with an anomaly

**Fig. 1:**
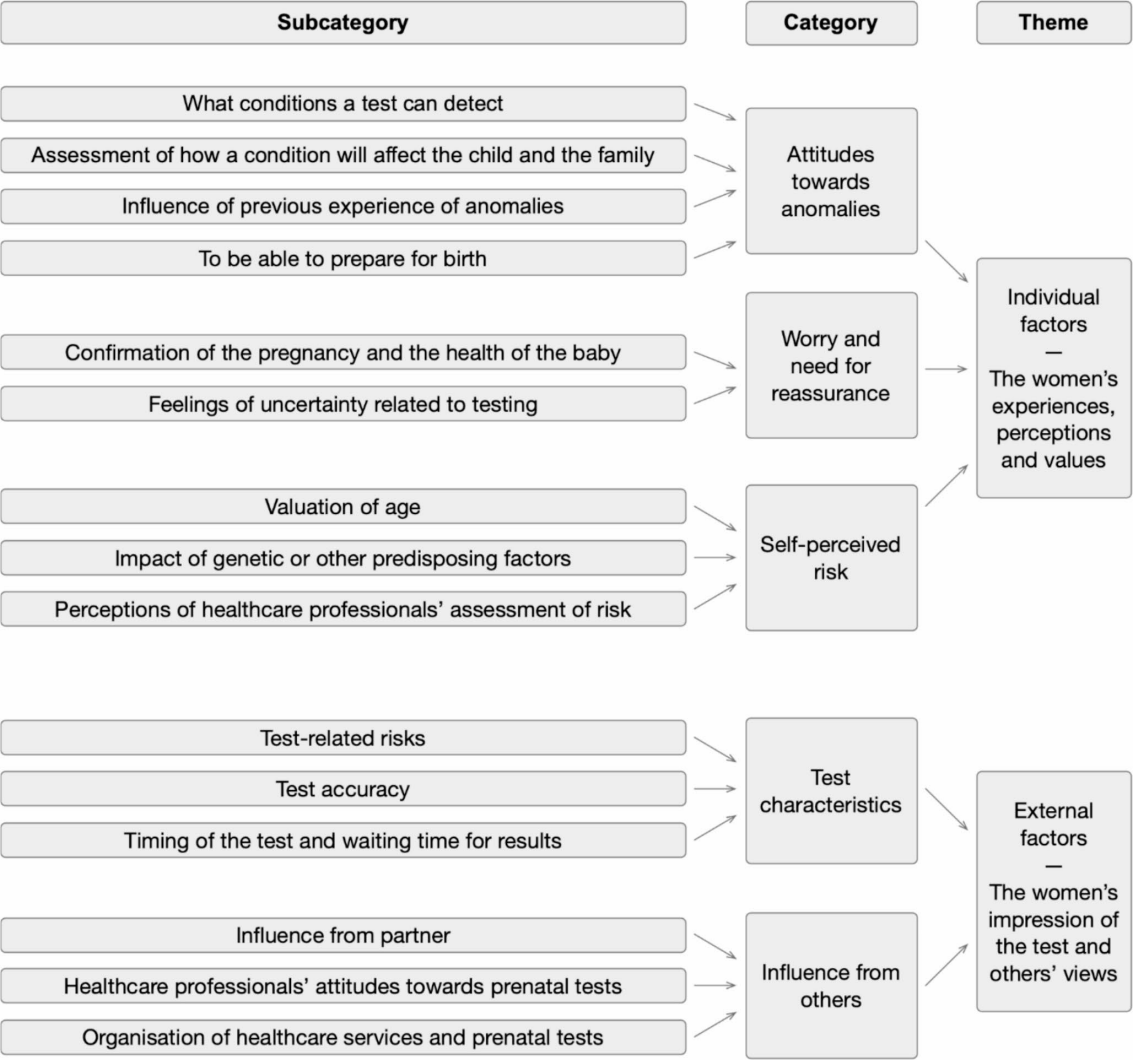
Category system describing the *individual* and *external factors* affecting the decision-making process

Two main themes emerged, with both individual and external factors influencing the decision-making process regarding PND (Fig. 1). The theme ‘Individual factors – The women’s experiences, perceptions and values’ had three categories (*‘Attitudes towards anomalies’*,* ‘Worry and need for reassurance’*,* ‘Self-perceived risk’*). The theme ‘External factors – The women’s impression of the test and others’ views’ had two categories *(‘Test characteristics’*,* Influence from others’).*

### Theme 1: Individual factors – the women’s experiences, perceptions and values

#### Attitudes towards anomalies

Some women expressed that they would want to test for as much as possible with no distinction between conditions. They wanted to gain as much information as possible about their fetus, but without posing any risks to the pregnancy. For others, their perception of CAs and other conditions played a substantial role in the decision-making process. *What conditions a test can detect* and the perceived severity of these conditions, influenced their decision-making process. What was considered a severe condition that motivated testing varied. Insufficient information (for women who had had their first visit with their midwife) and a lack of knowledge about conditions screened for made it harder for some to make decisions about testing.

*“I personally feel that we’ve not received a lot of information and I think this is why it’s been hard for us*,* or rather hard to decide what we should do [….] say it’s a test to find chromosomal disorders*,* well what would that mean? How does such a child function*,* or what implications does this kind of disorder have?” – Woman #8*,* age 27*,* nulliparous*.

Women’s *assessment of how the condition will affect the child and the family* was often a major factor when discussing how they felt about having PND for different conditions. The expected quality of life for the child was an essential factor in women’s decision-making process.

*“If the child were to be seriously ill*,* then what kind of life is one giving the child?” – Woman #17*,* age 32*,* parous*.

Conditions that would not cause suffering for the child and that could be consistent with a good quality of life, were often seen as more manageable and therefore not necessary to test for. Women also considered the possible negative effect on siblings (such as less time with parents) if the child had disabilities or health problems. Nulliparous women not interested in PND in their first ongoing pregnancy, admitted that they might feel differently in a subsequent pregnancy because of the effect a sibling with an anomaly might have on their first child. For less severe conditions, some women reasoned that siblings would adjust and the family would adapt.

For some women, the effect a child with an anomaly might have on their work and social life was an important factor when deciding about PND. They were worried that such a child would prevent them from living a full and happy life.

*“My job is really important to me. I’ve studied for a long time and put a whole lot of time into it. I love my job [….] both me and my partner live very active lives where we still have a lot of freedom*,* having children hasn’t changed this very much [….] should it become limiting for me*,* maybe I wouldn’t be*,* I don’t know how happy I would be anymore. I don’t think for a second that I would love this child less. But if I could choose to not live the rest of my life with a severely handicapped child*,* I would do it.” - Woman #2*,* age 33*,* parous*.

The prospect of a child who would not grow up and become independent worried some women. They were not sure they could cope with that lifelong responsibility, which could motivate both having PND and considering TOP if an anomaly was diagnosed.

*Previous experience of anomalies* or children with disabilities influenced the perception of what life with an affected child could be like. For some women, personal experience among family or friends made them more inclined to have PND, while others felt they could cope with the situation and that it would be worth the love the child would bring.

*“Among our relatives*,* we have a child with a chromosomal disorder and now we can’t imagine a life without him*,* so I think it would be the same here. In other words*,* you would adjust and just take it as it is. Love your child*,* make the best of it.” – Woman #11*,* age 35*,* parous.*

Women with previous experience from work with disabled children, reflected on how the severity of the condition affected quality of life and the need for assistance in daily life. One woman working as a personal assistant to children with disabilities, described a sense of security, knowing that the social welfare system in Sweden would provide help if needed. Yet she also acknowledged that her insight into the struggles of daily life could cause sadness if having a disabled child herself.

Women who would not consider or were undecided about undergoing a TOP in case of an anomaly, could still see a value in PND *to be able to prepare* for the birth of a child with special needs or health problems. Knowledge about the condition before birth could give them a chance to obtain information to better prepare for a life with an affected child.

*“It’s still probably good to know some things*,* because then one can prepare in a different way for when the baby arrives.” – Woman #4*,* age 27*,* nulliparous*.

#### Worry and need for reassurance

Women expressed a need for *confirmation of the pregnancy and the health of the baby*. Some wanted to undergo a CUB test because they wanted an early ultrasound and see the fetus. To wait for the second-trimester ultrasound, which is offered to all pregnant woman in Sweden, felt too long. Women described how it was hard for their partner to feel involved when the pregnancy was not yet visible. An ultrasound where both the woman and her partner could see the fetus made the pregnancy more real and was reassuring if fetal viability was confirmed, especially for women who had not yet felt pregnancy symptoms or fetal movements.

*“And the other partner can be made more involved*,* and in terms of myself*,* maybe also really understanding*,* yes – there actually is a life in there*,* even if it feels kind of unreal*,* so it’s really hard to imagine.” - Woman #8*,* age 27*,* nulliparous*.

Some women expressed that being able to get pregnant did not necessarily lead to a live-born baby, acknowledging that a lot could happen in 9 months. To rule out at least some conditions through PND could decrease worry and be reassuring. For women who had had a previous miscarriage, missed abortion or previous experience of fetal anomalies, a reassuring early ultrasound was especially important.

For some women, reasons for declining testing were *feelings of uncertainty related to testing*, such as that of a non-diagnostic probability assessment, e.g. the CUB test, and uncertainty about how to handle the results. Having a CUB test felt like risking unnecessary anxiety to some who had decided not to have an invasive test due to the associated risks. Waiting for the result was also mentioned as a period of increased anxiety.

*“I’ve also read about people who have written about it*,* who’ve taken this CUB test and got really high-risk numbers and then have been really worried during the entire pregnancy and then a healthy baby arrived. It’s really good that it was a healthy baby*,* but all the worry during the pregnancy*,* I don’t think it’s worth it.” – Woman #11*,* age 35*,* parous*.

#### Self-perceived risk

The self-perceived risk (i.e. the woman’s perception of her own risk) of expecting a fetus with a CA or other anomaly was described by pregnant women as a factor affecting their decision to undergo PND. Women with a low self-perceived risk did not feel a need for PND, while women with a higher self-perceived risk were more prone to undergo PND. Women were aware that with increasing age comes an increasing probability of CAs, and *valuation of age* was a major factor when assessing self-perceived risk. Older women, who in a previous pregnancy had not had PND, felt differently when now pregnant at their current age. Some also said they were now provided more extensive information about PND and experienced a different attitude from healthcare professionals. Younger women did not feel a need to undergo PND in their current pregnancy, but admitted that had they been older, they would have felt more inclined to have tests.

*“I think that if I had been older*,* then I would probably have checked*,* because I know there are more risks then.” – Woman #17*,* age 32*,* parous*.

Some women expressed that if everything looked normal at routine check-ups during pregnancy, they did not worry. Others described an *impact of genetic or other predisposing factors* causing an increased probability of a child with a CA, that if present in the parents, could motivate PND. The lifestyles of the pregnant women and partners were seen as possible predisposing factors, as well as their previous medical history and possible genetic conditions. Women with no family history of CAs were less inclined to have PND.

*“Since I don’t have it in my own family*,* I don’t think I would – nah*,* then I would probably choose not to in fact*,* and not do the test. It’s different if there had been a lot way back in the family*,* maybe then it would have felt like I had to.” – Woman #3*,* age 35*,* parous*.

The self-perceived risk was also affected by what healthcare professionals conveyed and the women’s *perceptions of healthcare professionals’ assessment of risk*. Women trusted healthcare professionals to let them know if there were reasons to worry or to have a test. The women thought the midwife made a professional assessment of the woman’s need to have PND. They expected to be given that information and if the midwife implied a higher probability, they would consider testing. To not be offered or recommended a test was perceived as a confirmation that the healthcare professional had made an assessment that their individual probability was low and testing not necessary.

*“In that case*,* I think that the risk or chance is really small then and if there was anything that they thought should be checked out*,* well then they would have told me.” - Woman #22*,* age 33*,* nulliparous*.

### Theme 2: External factors – the women’s impression of the test and others’ views

While individual factors play an important role in the decision-making for many women, external factors such as test characteristics and influence from others can be just as influential.

#### Test characteristics

The characteristics of a test affect how women regard the test. *Test-related risks* were a major factor, many times a dealbreaker, for women when deciding about PND. There was a clear preference for risk-free tests. Decisions to undergo tests with no risks seemed less difficult to make. Women who were otherwise in favour of having PND were often hesitant to have a test with a risk of miscarriage.

*“I don’t want to take any tests where there is a risk for miscarriage.” - Woman #20*,* age 26*,* nulliparous*.

For some, this resulted in not having PND since they already had decided not to have a subsequent invasive test if a screening test indicated an increased probability. Others chose to have a screening test even though they were hesitant to have invasive tests associated with risks.

In general, women were less positive about tests that had a high percentage of false positive or false negative results. For some women the *test accuracy* was an important factor and they were not interested in tests with uncertain results. Not knowing if a result was correct could cause worry, especially if the test indicated an anomaly, thus making decisions concerning the pregnancy difficult. Women found the results from a probability assessment, such as CUB, difficult to interpret as they would not provide a clear answer. Those who would not consider an invasive test due to the increased risk of miscarriage, often found decision-making regarding screening more complicated than women open to having an invasive test to verify a diagnosis.

*“You take the test to be sure*,* so if the test is not certain or not accurate to*,* well 99%*,* then there is probably no point in taking the test.” – Woman #6*,* age 27*,* nulliparous*.

The *timing of the test and waiting time for results* were other characteristics affecting women’s decision-making. For some women, early testing and short waiting time was desired in an attempt to ease their worry. The time from test to result was mentioned as a period of increased anxiety with time to worry about what the results might show. Information and knowledge of the standard procedure, including the expected time from test to result was appreciated, as it helped relieve some of the worry.

*“Just the waiting makes me so nervous and then it just means that you go around and worry and probably get a bunch of ideas in your head*,* everything that is wrong*,* even if like there maybe isn’t anything.” – Woman #18*,* age 28*,* nulliparous*.

Both timing of the test and time from test to result was important for women who could consider TOP following a diagnosis of a fetal anomaly. To these women, the time limit for TOP without a special permission (18 gestational weeks) was one reason for wanting early testing. Another reason was their feeling that the longer the pregnancy advanced, the more real it would become, especially with awareness of fetal movements. Having had time to bond with the unborn child would make the decision to have a TOP emotionally harder, as would having told people about the pregnancy both privately and professionally.

#### Influence from others

Women mentioned *influence from their partner*, from healthcare professionals and how PND was offered, as factors affecting their decisions. Some women stressed that decisions regarding their bodies, including the pregnancy, were their own. However, most women wished to receive information regarding PND followed by time to reflect with their partner before making a joint decision. Some also described how their decision had been influenced by their partner’s attitude towards PND.

*“I would probably prefer to get the information and then discuss it with the person I live with. What thoughts*,* what we want and so on.” – Woman #23*,* age 31*,* nulliparous*.

*Healthcare professionals’ attitudes towards PND *could affect women in both directions, i.e. to pursue or abstain from testing. Some women admitted feeling pressured to have a test if it was presented by healthcare professionals as something you should do. Others described how a judgmental attitude towards testing and TOP could dissuade them from testing. One woman even described having cancelled her already booked appointment for a CUB test after her midwife had questioned it.

*“So maybe you don’t follow your heart*,* and instead be influenced by how the medical staff… what the particular person you meet thinks.” – Woman #2*,* age 33*,* parous*.

*The organisation of healthcare services* and the simple fact that PND is offered by the public antenatal care was interpreted as a recommendation by some women. By offering CUB at a subsidised cost only to specific age groups, e.g. women over 35, an increased probability of fetal anomalies is indirectly emphasised, making some older women feel compelled to have the test. Younger women, who due to age, were not offered CUB, often ended up not having a test even though they would have liked to. To some, the option to travel to another region and pay the full cost for the test privately was a hinder.

*“I think I would see it as a recommendation*,* because I am so unsure myself*,* even if it is an offer.” – Woman #13*,* age 26*,* nulliparous*.

## Discussion

Pregnant women’s decision-making process regarding PND is multidimensional, affected by both individual and external factors. Previous research has studied some of the aspects on the decision-making process. With a broader perspective of the multidimensional decision-making process, this study provides a deeper understanding of how the various factors interplay and influence the decision-making. This is of importance for healthcare professionals offering PND services. It highlights the need for providing accurate and well-balanced information about test methods and conditions tested for, since these are factors influencing women’s decisions. Assistance from healthcare professionals to assess the individual likelihood of expecting a disabled child, based on background characteristics and family history, is of help when women make decisions about PND. In addition, healthcare professionals need to be aware how their attitudes can affect the women’s decision-making process.

### Worry, self-perceived risk and influence from healthcare

The choice to undergo PND can be driven by anxiety, the wish to know as much as possible about the pregnancy and to be reassured that the fetus is healthy; findings well in line with previous reports (Crombag et al. 2013; Miltoft et al. 2018; Nykänen et al. 2017; Ternby et al. 2015, 2016). Bakst et al. (Bakst et al. 2019) suggest that decisions regarding PND might be more influenced by social and emotional factors then by the actual probability. We found that such factors influence the decision, but for some women the self-perceived risk was an important reason for choosing or not choosing PND (van Bruggen et al. 2018; Crombag et al. 2016a; Ternby et al. 2016). Other studies indicate that women with advanced maternal age or genetic risk factors are more likely to undergo prenatal testing (van Bruggen et al. 2018; Di Mattei et al. 2021; Ternby et al. 2015, 2016), which might be due to their self-perceived risk and that these women are offered PND to a greater extent. We found that the offer of PND in public healthcare might be perceived as a recommendation or as part of routine antenatal care, and therefore something one should do. This has also been highlighted previously (Lewis et al. 2016a; Skirton and Barr 2007; Ternby et al. 2016; Ternby et al. 2015). Information and counselling about PND should be neutral and non-directive (Dondorp et al. 2015), but undue influence from healthcare professionals on women’s decision-making exists (Damman et al. 2023; van der Steen et al. 2019; Ukuhor et al. 2017). The way healthcare professionals present the tests characteristics and provide the individual probability status can affect how pregnant women perceives their own probability and need for PND. Women may assume that their midwife makes an individual assessment of her need for PND based on her background characteristics. Therefore, when the midwife does not offer or recommend a woman to undergo a test, it might by the woman be interpreted as an unspoken confirmation that the midwife has assessed her individual probability as low and testing is not necessary. Healthcare professionals need to be aware that what they say, but also what they do not say can influence women’s decisions.

### Perceived social pressure, attitudes to termination of pregnancy and risk of miscarriage

There is a risk that women’s decisions regarding PND and possible TOP may be affected by perceived social pressure (van Bruggen et al. 2018; Ngan et al. 2020). Trust or mistrust towards the medical establishment may have an effect on decision-making, sometimes with the assumption that healthcare and society in general regard TOP as the “right” decision if a CA is diagnosed (Allyse et al. 2015; García et al. 2008). Previous studies have found that women who had a more positive attitude towards TOP and would consider TOP in the event of an abnormal result, were more likely to use prenatal tests (Bakst et al. 2019; Crombag et al. 2016a; Di Mattei et al. 2021; Ternby et al. 2016) and thereby to accept the increased risk of miscarriage associated with invasive tests. The present study indicates that for some women PND can be a way to prepare to have a child with a CA, which has previously been reported (van Bruggen et al. 2018; Di Mattei et al. 2021; Miltoft et al. 2018; Ternby et al. 2015, 2016). For these women, a test that includes a risk of miscarriage is often not an option (Lewis et al. 2014; Miltoft et al. 2018), making test characteristics a deal breaker. Other women, who do not consider TOP as an alternative, choose not to have any tests (Lewis et al. 2014, 2016b; Ternby et al. 2016). To some expectant parents, the CUB test is seen as an extra opportunity to see the fetus (Nykänen et al. 2017) and is not necessarily seen as PND for CAs. Offering these women a first-trimester ultrasound without the biochemistry and probability assessment might be a better solution as they could opt out of screening for CAs but still get the ultrasound.

### Test characteristics and the availability of tests

NIPT is the preferred test for pregnant women in both low- and high-risk pregnancies (Di Mattei et al. 2021). In the present study, test-related factors such as risks, accuracy, reliability and timing of the tests are important when women make decisions about PND, congruent with previous reports (Di Mattei et al. 2021; Hill et al. 2016; Lewis et al. 2016a; Lewis et al. 2014; Miltoft et al. 2018; Ternby et al. 2016). However, the uncertainty of a test result from a non-diagnostic test, such as CUB or NIPT, or the anxiety caused by waiting for the results or by receiving a false positive result, were mentioned as reasons to decline testing, also in line with previous findings (Crombag et al. 2013; Crombag et al. 2016b; Crombag et al. 2016a). The availability and ease of having a test can also facilitate or hamper the option to have a test. In Sweden, most public healthcare services are subsidised, except for NIPT which is offered subsidised only in the case of an increased probability of a CA. Moreover, how primary screening with CUB is offered differs among regions (in some to everyone, in some based on age, in some not at all). To some women, the latter was the main reason not to undergo PND even though they wanted to, since this would cost them both extra time and money to travel to another region and pay the full price. The effect the cost of tests can have on decisions about PND has previously been shown (Allyse et al. 2015; Bakker et al. 2012; Damman et al. 2023). Some have argued that the cost may not affect the final decision, but make women aware that the test is optional and not a routine (van Bruggen et al. 2018; Crombag et al. 2016a; Damman et al. 2023).

### Quality of life and impact on the family

When considering PND and possible TOP, the expected quality of life of a disabled child is an important factor for many women (van Bruggen et al. 2018; Lewis et al. 2016a; Miltoft et al. 2018; Ngan et al. 2020). What was considered a severe condition varied, often due to previous experiences and attitudes towards anomalies, which has previously been shown to affect decisions concerning PND (Damman et al. 2023; Lewis et al. 2014; Miltoft et al. 2018). Having previous experience of someone with an anomaly in your vicinity can decrease decisional conflict related to decisions about PND, as you have experiential knowledge to base the decision upon. For women who have a child with an anomaly, or know someone with an anomaly, there might also be an element of guilt or perceived disapproval from others involved when making decisions about PND (Bryant et al. 2005). Moreover, a person’s view on parental responsibilities and decisions about PND can be context-dependent as indicated by the subcategory “How the condition will affect the child and the family”. The expected physical and emotional impact that a disabled child would have on the expectant parents and their family seems to affect the decision-making regarding PND (van Bruggen et al. 2018; Garcia et al. 2022; Lewis et al. 2016a; Miltoft et al. 2018) and TOP (Garcia et al. 2022; Ngan et al. 2020). Hence, the decision to accept or decline PND might vary for one person depending on the circumstances at that point in life and if they have previous children or not (Garcia et al. 2022). Even though the decision regarding PND is the woman’s own, many women seem to be influenced by their partner’s opinions and choose to make the decision as a couple (Damman et al. 2023; Laberge et al. 2019; Miltoft et al. 2018; Sahlin et al. 2016). Decisions about PND are many times difficult, and without knowledge about the conditions tested for, the decision can be even more difficult. Information about medical, cognitive and social consequences of the conditions tested for could enable expectant parents to make an informed decision since it gives them better knowledge of how a child with a specific condition could be affected, and how it could affect the parents and their family (Lewis et al. 2014).

### Strengths and weaknesses

As the recent review by Di Mattei et al. (Di Mattei et al. 2021) did not include qualitative studies, the present study adds to the knowledge from quantitative research by exploring pregnant women’s views in more depth. One strength of the study is that most of the participants were approached and included in early pregnancy, before they had met with a midwife and received information regarding PND and conditions screened for. Thus, it is unlikely that their opinions were influenced by information from healthcare professionals. Of course, those who had been pregnant before might be influenced by previous discussions about PND and CAs. Another strength is the participants’ different educational levels, age and parity, which increases the transferability of the findings. However, due to language barriers, non-Swedish speaking women were not included, which is a limitation. The research team involved in analysis and interpretation had different professional backgrounds relevant to the research question, giving complementary perspectives when interpreting the data. No diverse cases or minor themes were left out from the final category system. As with any qualitative research, no claims of generalisability can be made, but the results from the present study are congruent with previous quantitative and qualitative research and they give us a picture of what factors influence the decision-making process regarding PND.

## Conclusions and clinical implications

Pregnant women’s decision-making process regarding PND is multidimensional, affected by both individual factors like experiences, perceptions and values as well as external factors like test characteristics and influence from others. Well-balanced information about both test methods and factors affecting the probability for CAs as well as about conditions tested for and how it might affect the child and the family are equally important to facilitate an informed choice. Future research on informed choice and knowledge among expectant parents would benefit from including not only knowledge about prenatal tests, but also more information and knowledge about the conditions tested for. It is important that healthcare professionals who counsel pregnant women are aware of how their attitudes and demeaner might influence pregnant women’s decisions. They need to be aware that what they do say, but also what they do not say, influences women’s decisions.

## Electronic supplementary material

Below is the link to the electronic supplementary material.

Supplementary Material 1

## Data Availability

The data that support the findings of this study are available from the corresponding author upon reasonable request.

## Abbreviations

PND: Prenatal diagnosis (including both screening tests and diagnostic tests)
CUB test: Combined ultrasound and biochemistry test
NIPT: Non-Invasive prenatal testing
CA: Chromosomal anomaly
TOP: Termination of pregnancy
