# Supplementary material for: Factors influencing pregnant women’s decision to accept or decline prenatal screening and diagnosis – a qualitative study
Source: J Community Genet. 2024 Nov 1;15(6):711–21. doi: 10.1007/s12687-024-00746-3 (PMC11645329; doi:10.1007/s12687-024-00746-3)
Supplement: Supplementary file 1 — Supplementary Material 1 [file 12687_2024_746_MOESM1_ESM.doc]

**Interview guide**

| THEME | QUESTIONS |
| --- | --- |
| 1) Information that is relevant for expectant parents being able to make informed choices about prenatal diagnosis. | - What are your thoughts about prenatal diagnosis?  - Describe the grounds on which you make decisions about prenatal diagnosis.  - What information do you need in order to make a decision?  - What information about the conditions that are tested for, do you think is important/relevant to receive prior to prenatal testing.  - When would you prefer to receive information about prenatal diagnosis and the conditions that can be identified – prior to testing or when one receives a result showing high risk? |
| 2) How does it feel to make decisions about prenatal diagnosis and what factors play a role in the decision? | - How difficult do you think it is to make a decision about declining or undergoing prenatal diagnosis?  - What factors play a role in the decision?  - If the test is invasive or not?  - How early in the pregnancy the test can be taken?  - How long it takes to receive an answer?  - What can be detected?  - The test’s accuracy/inaccuracy, false positive/false negative results? |
| 3) Professional demeanour in conjunction with receiving information about prenatal diagnosis | - What do you think is important in the demeanour of healthcare professionals when you receive information about prenatal diagnosis? |
| 4) Expectant parents’ attitudes and reflections about Down syndrome. | - How do you regard having a child with a disorder?  - What are your thoughts about Down syndrome?  -How do you think having a child with Down syndrome would affect you?  -How do you think it would affect your relationship, your family, your career etc.? |

Manuscript submitted to: Journal of Community Genetics

Title of manuscript: Factors influencing pregnant women’s decision to accept or decline prenatal diagnosis – A qualitative study

Authors: Ellen Ternby^1^, Ove Axelsson^1,2^, Charlotta Ingvoldstad Malmgren^3-5^, Susanne Georgsson^6^

Corresponding Author:

Ellen Ternby

Department of Women’s and Children’s Health, Uppsala University

Email: ellen.ternby@uu.se
